# Splenic artery transposition for hepatic arterial supply in living donor liver transplantation

**DOI:** 10.12669/pjms.39.1.6351

**Published:** 2023

**Authors:** Kaleem Ullah, Abdul Wahab Dogar, Shams-ud-Din, Hafiz Bilal

**Affiliations:** 1 Kaleem Ullah, FCPS. Pir Abdul Qadir Shah Jeelani Institute of Medical Sciences, Gambat, Sindh, Pakistan; 2 Abdul Wahab Dogar, FCPS. Pir Abdul Qadir Shah Jeelani Institute of Medical Sciences, Gambat, Sindh, Pakistan; 3 Shams-ud-Din, FCPS. Pir Abdul Qadir Shah Jeelani Institute of Medical Sciences, Gambat, Sindh, Pakistan; 4 Hafiz Bilal, FCPS. Pir Abdul Qadir Shah Jeelani Institute of Medical Sciences, Gambat, Sindh, Pakistan

**Keywords:** Living donor, Splenic artery, Liver transplantation, Transposition

## Abstract

**Objective:**

To determine the safety and outcome of splenic artery(SA) transposition in extra-anatomic hepatic arterial reconstruction (HAR) in living donor liver transplantation(LDLT).

**Methods:**

We retrospectively compared the outcome of the ten liver recipients who underwent HAR with the transposed splenic artery (SA group) with a matched cohort of 40 recipients who underwent HAR with the standard hepatic artery (HA group) between March, 2019 and December, 2020 at liver transplantation department, Pir Abdul Qadir Shah Jeelani Institute of Medical Sciences, Pakistan. The comparison of recipients’ and donor demographics, operative and graft characteristics, post-operative labs, Doppler ultrasound(USG) findings, and complications, along with 30-day mortality, and 1-year survival were reported for both groups.

**Results:**

The mean age of patients in the SA group was 42.80±7.510 and in the HA group was 43.73±8.171 years. The common indication of LDLT was viral hepatitis in both groups. The operative duration was longer in the SA group (597.50±41.3156 min) than in the HA group (530.75±66.502 min) with a significant p-value= 0.004. Similarly, blood loss was also more in the SA group (1635±226.139 ml) than in the HA group (1477.50±270.316 ml) (p-value= 0.096). The incidence of biliary and vascular complications, early allograft dysfunction, acute cellular rejection, 30-day mortality, and 1-year survival were comparable in both groups. Post-operatively splenectomy was not needed in any SA group recipients.

**Conclusion:**

The SA is easily approachable, suitable, and safe for HAR in the difficult situation of hepatic arterial flow inadequacy during LDLT due to its appropriate length, and good blood flow.

## INTRODUCTION

Living donor liver transplantation (LDLT) has become the modality of choice for treating patients with end-stage liver disease, hepatocellular carcinoma, and hepatic metabolic disorders.^1^ Unfortunately, the expansion of LDLT increases the likelihood of encountering various vascular complications, most importantly hepatic arterial complications.^2^

Hepatic arterial reconstruction(HAR) is one of the crucial and difficult steps in LDLT, which always demands skilled and meticulous surgical techniques.^3^ In LDLT the narrow lumen and short stump of graft artery and the lumen discrepancy between the graft and recipient arteries, make HAR a challenging and difficult task compared to HAR in deceased donor liver transplantation(DDLT).^2^ An appropriate recipient hepatic artery(HA) having adequate blood flow, length, and caliber along with optimal surgical techniques are the key factors for successful HAR.^3^

For a variety of reasons i.e., sub-intimal dissection, inadequate blood flow, and due to short length, the HA or its branches may not be suitable for anatomic HAR. In such difficult situations, alternative arterial inflow techniques are practiced which is called extra-anatomic HAR. Several techniques of extra-anatomic HAR had been reported. In DDLT frequently the donor iliac artery conduit is utilized for arterial inflow from the aorta.^3^ But due to the non-availability of this option in LDLT, various transposed arteries like the left gastric artery(LGA), right gastric epiploic artery(RGE), gastro-duodenal artery(GDA), splenic artery(SA), or even saphenous vein conduit from aorta had been reported for HAR with variable results.^3^-^6^

Various extra-anatomic HAR techniques are practiced when native recipient HA is not suitable, but still, no consensus is achieved on a single technique. Few authors have reported the outcome of SA utilization for HAR in DDLT.^7^ However, there is a paucity of data on the utilization of SA for HAR in LDLT. We have previously reported a case of HAR with transposed SA in an LDLT recipient.^8^ Since then, the utilization of SA for extra-anatomic HAR is been our preferred choice, when the native recipient HA is unsuitable. Here, we aimed to review and share our experience and outcome of 10 LDLT recipients who underwent HAR with transposed SA.

## METHODS

This single-center retrospective cohort study was conducted at Pir Abdul Qadir Shah Jeelani Institute of Medical Sciences, Gambat, Pakistan. A total of 320 LDLT procedures were performed between March 2019 and December 2020, in a twenty-two months duration. These patients’ data was maintained prospectively on a computer-based database.

### Criteria for selection of study cohort:

The data of adult LDLT recipients were evaluated retrospectively to identify those who received SA grafts (extra-anatomic) for HAR. Out of all, a total of ten LDLT recipients’ received right lobe grafts and underwent HAR with transposed SA. For the comparative analysis, we compared the outcome of ten living donor grafts that underwent HAR with the transposed splenic artery (SA group, n = 10) with a matched cohort of forty right lobe grafts having HAR with the native hepatic artery (HA group, n = 40) in 1:4. Matching was done on basis of graft type and demographics (age, gender, and body mass index). Patients and donor demographic characteristics, operative parameters, post-transplant complications, operative, and post-op Doppler USG findings, 30-day mortality, and 1-year survival of both groups were compared. The study was carried out with the approval of the hospital’s Ethical Committee via Reference no: PASQJIMS/IRB/890.

As per our centers’ LDLT donor selection criteria, the donors were selected having an age range of 18 to 40 years, Body mass index (BMI) <25 kg/m^2^, no co-morbidities, normal baseline laboratory parameters, and a normal thrombophilia profile. Donor Tri-phasic computed tomographic scan for vascular and volumetric analysis, and a magnetic resonance cholangiography for biliary anatomy assessment was done. The recipient listing criteria include a Child-Turcotte-Pugh stage(CTP) score of C, Model for End-stage Liver Disease(MELD) score of ≥ 15, and patients with hepatocellular carcinoma (HCC) having tumor burden within UCSF criteria. The recipient’s upper age limit was 65 years. Pre-transplant imaging with contrast-enhanced CT scan was done in all recipients for vascular anatomy assessment. Routine pre-operative vaccination for encapsulated bacteria was done for all recipients as per protocol.

All the patients included in this study received right lobe grafts with or without middle hepatic vein (MHV) reconstruction. Pediatric-age recipients and those who received left lobe grafts, recipients whose indication of transplantation was acute liver failure/ acute on chronic liver failure, recipients who received jump grafts for portal vein thrombosis, and re-transplanted cases were excluded from the study. Informed written consent was taken from all donors and recipients for procedure and research purposes.

The piggyback technique was employed in all patients. The recipient’s HA was the artery of choice for anatomic HAR. Right or left HA, the HA proper, or common HA were used for anatomical HAR, depending upon caliber, flow, and length of the artery. Any undue angulation or arterial redundancy was also avoided. For the grafts with two HAs, the non-dominant graft artery was reconstructed only if it was quite large or if inadequate backflow was found after the reconstruction of the dominant artery. In cases where native recipient HA was not found suitable for HAR, we targeted the SA for HAR.

### Surgical technique of SA to HA anastomosis:

Before proceeding with SA utilization, the CT scan features of SA were re-evaluated per-operatively. Various parameters like SA size, course, tortuosity, aneurysmal dilation, and relationship with the pancreas, all were assessed carefully on the CT scan. Also, the intraoperative field was judged for consideration of SA.

**Fig.1:**
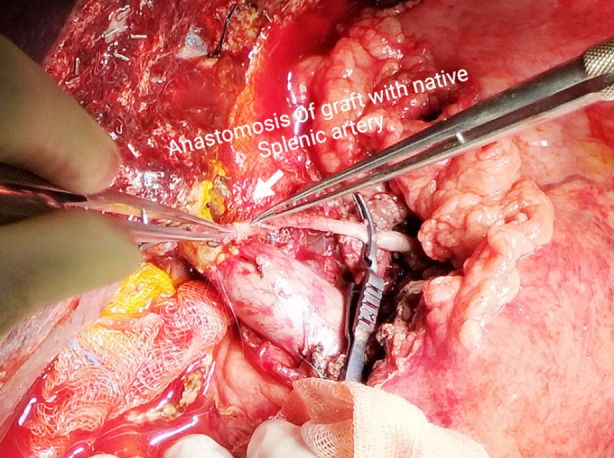
Showing graft artery anastomosis with recipient SA.

The SA transposition began with entry to the lesser sac with the division of the gastro-colic ligament. The pulsation of SA was checked at the upper border of the pancreas and then supra-pancreatic dissection was started with monopolar and bipolar cautery. Three to four small pancreatic branches were ligated and divided, and the dissection was continued till the SA was identified. SA was then encircled with a vascular sling and a bulldog clamp was applied proximally on SA. After getting the appropriate length of SA, the distal end was ligated twice and divided. Before the division of SA, portal flow adequacy was confirmed on the Doppler examination. An appropriate length of SA stump was acquired, rotated, and transposed towards the hepatic hilum. The extra length of SA was then trimmed and the margins were refreshed with a sharp scissor. Any redundancy and angulation were taken care of. The excess of neuro-lymphatic tissues around the adventitia was also trimmed. The backflow from the graft HA was prevented with a microvascular bulldog clamp. The anastomosis was performed with polypropylene 8-0 suture, in an interrupted end-to-end fashion under 3.5 magnification loupes (Fig.1). After the anastomosis, an on-table Doppler USG was performed for confirmation of patency and flow.

### Anticoagulation and Immuno-suppression protocol:

Prophylactic anticoagulation was done with low molecular weight heparin (Enoxaparin sodium) with a dose of 1 to 2 mg/kg/body weight. Anticoagulation usually started on the 2^nd^ post-operative (POD) day and onward and continued for seven days except in patients having an INR >2, platelet count of <30*10^3^/L, and those with difficult hemostasis. On the 7^th^ POD oral aspirin 75mg once daily was started and continued till six months while enoxaparin was discontinued on the 8^th^ POD.

Post-operatively dual immunosuppression oral tacrolimus and prednisolone were given. Prednisolone was tapered and stopped till the end of three months while oral tacrolimus was continued as maintenance immunosuppression as per protocol. The detailed immunosuppression protocol is described somewhere else.^9^

### Post-transplant Doppler protocol:

To keep an eye on arterial complications after the transplant, daily Doppler ultrasound (USG) was performed for the first five days continuously and then at the time of discharge (i.e. usually on the 10^th^ POD). After that, the Doppler USG was repeated on the follow-ups i.e. fortnightly for 1^st^ month and then at the 3^rd^, 6^th^, and 12^th^ months. Arterial complications were suspected when Doppler USG revealed abnormalities in the flow and velocity associated with elevated liver enzymes. For any abnormal Doppler findings, a CT angiography was done for further confirmation.

### Statistical Analysis:

Differences between the two groups were evaluated using parametric and nonparametric tests. Qualitative variables were analyzed using the Fisher test or chi-square test, while quantitative variables were analyzed using the student t-test, or in the case of normal distributions, the Mann–Whitney U Test Was used. All statistical analyses were performed using IIBM SPSS Version 21. A p-value of <0.05 was set as significant.

## RESULTS

In ten cases, when HA arterial flow was inadequate in the recipient per-operatively, SA was utilized for inflow. The mean age of patients in the SA group was 42.80±7.510 years and 43.73±8.171 years in the HA group. The most common etiology of chronic liver disease was viral hepatitis in both groups. No significant differences were recorded in both the groups while comparing recipients’ demographics, etiology, the severity of disease i.e. CTP and MELD scores, HCC, and history of previous trans-arterial chemoembolization (Table-I).

**Table-I:** Comparison of donor parameters, recipients’ demographics, operative parameters, and graft characteristics.

Parameters	SA group n=10	HA group n=40	p-value
Recipient mean age (years)	42.80±7.510	43.73±8.171	0.747
Mean BMI (Kg/m2)	21.96±3.897	23.30±4.735	0.413
***Recipient Gender***			0.053
Male	5(50%)	32(80%)	
Female	5(50%)	8(20%))	
***Etiology***			0.646
HBV	2(20%)	9(22.5%)	
HBV, HDV	2(20%)	15(37.5%)	
HCV	4(40%)	12(30%)	
Cryptogenic	2(20%)	4(10%)	
HCC	1(10%)	4(10%)	1.00
TACE history	1(10%)	4(10%)	1.00
DM	0	1(2.5%)	0.614
HTN	0	2(5%)	0.470
***Child Score***			0.373
Class A	1(10%)	1(2.5%)	
Class B	2(20%)	15(37.5%)	
Class C	7(70%)	24(60%)	
***Mean MELD Score***	18.98±5.867	19.40±4.986	0.798
***Donor parameters***			
Donor mean Age(years)	23.40±5.147	22.33±5.111	0.555
Donor mean BMI (kg/m^2)^	21.576±3.364	21.050±2.963	0.628
Donor mean LAI	10.460±5.282	10.452±4.969	0.977
***Recipient’s Operative parameters***			
Mean GRWR	1.122±0.476	1.0804±0.284	0.744
Mean warm ischemia time (min)	27.30±7.105	28.50±8.651	0.687
Mean cold ischemia time (min)	9.70±4.084	9.03±4.526	0.656
Mean blood loss(ml)	1635±226.139	1477.50±270.316	0.096
Mean operation time (min)	597.50±41.315	530.75±66.502	0.004
PRBCs transfusion	6(60%)	22(55%)	0.776
***Graft Type***			0.076
RLG Without MHV	9(90%)	21(52.5%)	
RLG with full MHV	1(10%)	7(17.5%)	
RLG with Partial MHV	0	12(30%)	

BMI, Body mass index; BMI, body mass index; HCV, hepatitis C virus; HBV, hepatitis B virus; HDV, hepatitis D virus; MELD, Model for End-Stage Liver Disease; HCC, Hepatocellular carcinoma; TACE, trans-arterial chemoembolization; DM, Diabetes; HTN, Hypertension; GRWR, Graft recipient weight ratio; PRBC, packed red blood cells; RLG, Right Lobe Graft; MHV, Middle hepatic vein; LAI, Liver attenuation index.

Various Donor parameters i.e., mean age, BMI, and mean liver attenuation index (LAI) were also comparable in both groups (Table-I). Similarly, various recipient’s operative parameters like mean cold and warm ischemia times were also comparable in both groups. The operative duration was longer in the SA group (597.50±41.3156 min) than in the HA group (530.75±66.502 min) and the comparison was a statistically significant p-value (0.004). Similarly, blood loss in the SA group was more (1635±226.139 ml) as compared to the HA group (1477.50±270.316 ml) with a p-value of 0.096. Details are given in Table-I.

Moreover, regarding the comparison of various recipient post-operative labs, the mean values of total bilirubin level, ALT, AST, and INR on the 7^th^ POD were comparable in both groups (Table-II). The hospital stay was also comparable in both groups.

**Fig.2:**
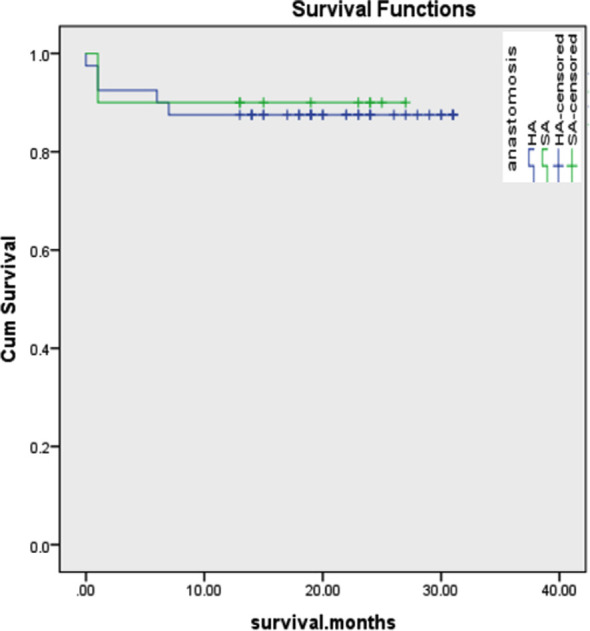
Post-transplant Kaplan meier survival analysis comparison between both groups (Minimum survival was 1-year).

**Table-II:** Comparison of mean Post-Operative laboratory parameters and post-op complications.

Parameters	SA group (n= 10)	HA group (n= 40)	p-value
***7th POD Mean* *Lab-parameter***			
Total Bilirubin(mg/dl)	2.050±0.680	2.156±1.607	0.840
AST(IU/L)	141.20±176.870	93.675±48.268	0.134
ALT(IU/L)	217.10±287.891	170.25±126.627	0.442
INR	1.3480±.208	1.485±0.456	0.362
***Complications***			
Early graft dysfunction	1(10%)	5(12.5%)	0.828
Splenic Infarction	1(10%)	00	0.043
Acute pancreatitis	00	00	00
Arterial thrombosis	00	1(2.5%)	0.614
Aterial stenosis	00	00	00
Portal vein thrombosis	00	00	00
Acute cellular Rejection	1(10%)	3(7.5%)	0.794
***Biliary complications***			0.10
Stricture	2(20%)	5(12.5%)	
Leak	1(10%)	0	
***30-day Mortality***	1(10%)	3(7.5%)	0.794

AST, Aspartate transaminase; ALT, Alanine transaminase; INR, international normalized ratio.

Regarding the complications directly related to the SA transposition technique i.e., splenic ischemia (infarction) was noted in a single patient in the SA group without clinically significant relevance. While splenic abscess and acute pancreatitis did not occur in any patient in the SA group. Incidence of biliary and vascular complications, re-operation, allograft dysfunction, Acute cellular rejection, and early 30-day mortality was statistically comparable between both groups(Table-II). The Doppler ultrasounds performed during operative and post-operative periods were normal in all SA group patients and their findings were comparable to the HA group (Table-III).

**Table-III:** Shows a comparison of the mean on the table and post-op resistive index (RI) of graft artery on doppler USG.

Time of Doppler USG	Mean RI of SA group recipients	Mean RI of SA group recipients	P-value
Operative Doppler	0.67±0.13	0.64±0.15	0.565
POD-1	0.69±0.17	0.71±0.12	0.667
POD-3	0.73±0.16	0.69±0.13	0.41
POD-5	0.70±0.19	0.64±0.17	0.334
At hospital discharge	0.63±0.14	0.68±0.12	0.259

Kaplan-Meier analysis (Fig.1) showed that the one year post-LT survival for the SA and HA Group was 90% and 87.5% respectively (log-rank p=0.831, minimum survival of one year). On follow-up, all the recipients having HAR with SA showed patent lumen and normal flow.

## DISCUSSION

Adequate arterial flow is mandatory for a successful LT outcome.^9^ While deficient arterial flow causes graft dysfunction, biliary complications, and graft failure.^2^,^10^ Conventionally, the graft artery is anastomosed with the recipient’s native HA.^9^ In situations of inadequate hepatic arterial flow, various other transposed arteries can be utilized for HAR.^4^-^8^

Literature had shown variable results regarding the utilization of the RGE artery for HAR. Wang et al. reported a 50% incidence of post-operative biliary complications in recipients who received RGE artery for HAR.^4^ However, Ahn et al. did not observe the same with the utilization of the RGE artery for HAR.^11^ We avoid the RGE artery for HAR, as we observed that the RGE artery has a small lumen with inadequate blood flow. And also we avoid LGA due to inadequate length for HAR.

In a few cases, we opted to use GDA for HAR but in the majority of cases, the GDA was not found suitable for HAR, either due to inadequate length/blood flow or due to intimal dissection extension into the GDA. It is noteworthy that the intimal dissection can very rarely extend into SA, as we observed it only in a single patient. In such cases, the supra-renal or infra-renal aortic conduit may be the preferable choice. But exposing the Infra-renal and supra celiac aorta can be quite challenging, especially with collaterals, adhesions, and in obese patients. Also, literature has shown an inferior outcome with venous conduit use due to a higher risk of thrombosis.^12^

The first report of HAR in DDLT using recipient SA was published by Figueras et al.^13^ The several technical advantages of utilizing the SA are easy accessibility, length adequacy, and good blood flow.^13^-^15^ We usually experienced good flow and superficial course of SA in cirrhotic patients, which made it feasible for HAR. In case of a diameter mismatch between splenic and graft arteries, the conning technique or end-to-side anastomosis can be tried. We did not encounter mismatch problems and we performed end-to-end anastomosis in all of our cases.

Pancreatitis secondary to peri-pancreatic tissue dissection, and splenic infarction are the potential complications secondary to SA diversion. Dokmak et al.^16^ attributed an increased risk of hemorrhage and pancreatitis to SA diversion. Although, we did not observe pancreatitis with SA diversion. However, in a single case, splenic infarction was observed sonographically without any evidence of splenic abscess and the patient recovered. Moreover, the spleen has got considerable supply from the short gastric vessels which supply the spleen in cases of SA diversion. Also, the immunological function of the spleen is not disturbed with SA diversion as confirmed by the experimental study.^17^

Regarding the operative blood loss, we observed a bit more blood loss in the SA group compared to the HA group. The reason may be that extra operative dissection which needed for SA mobilization. However, the comparison between both groups was not found statistically significant(p=0.096). This increased blood loss with SA transposition was also reported in another study.^7^ Similarly, the mean operative time was more in the SA group (597.50±41.315min) than HA (530.75±66.502 min). Llado et al. also reported more operative duration in cases having HAR with SA as compared to the standard technique.^7^

We also did not record any cases of early or late arterial thrombosis in any of our SA recipients (the minimum follow-up was 1-year). These results are comparatively better in comparison to other techniques.^18^,^19^

In this study, the early mortality and 1- year survival was comparable in both groups. Till the last follow-up, all patients of the SA group are alive except for a single early mortality secondary to respiratory complication and not related to the complication of SA diversion. On other hand, the literature has shown variable survival rates with the use of aorto-hepatic conduits .^3^,^20^-^22^ Few have shown optimal results 3 while many others have shown inferior results with the use of venous conduit from the aorta for graft arterial inflow.^20^-^22^. Hibi et al. strongly recommended the limited use of conduits because of inferior outcomes.^20^ Furthermore, conduits should be used only as a salvageable therapy.^22^

In the situation of hepatic arterial flow inadequacy, the native SA transposition has become our preferred alternative technique. We have reserved the other arterial transposition techniques and the use of conduits for circumstances where SA transposition may not be feasible or failed. We believe that this technique is under-utilized and transplant surgeons should consider this when the recipient’s native HA is unsuitable for HAR.

### Limitations:

One was the retrospective nature of this study and the second was relatively the small sample size. The statistical comparison between both the groups in our study was tough due to this small sample size. However, our main focus was to investigate the safety of this alternative arterialization technique in LDLT. In the future, we plan to continue practicing this technique and publish our results with larger sample size and longer follow-ups.

## CONCLUSION

Our results determined that in the situation of hepatic arterial flow inadequacy in LDLT, the use of the SA is a safe, effective, and practical alternative for extra-anatomic HAR. The SA is easily approachable and suitable for HAR due to its appropriate length, and good blood flow.

### Consent:

Informed consent was taken from all the participants.

### Authors’ Contribution:

**KU:** Wrote the manuscript and did the statistical analysis.

**AWD:** Designed the study and did the final editing of the manuscript.

**SUD:** Takes the responsibility and is accountable for all aspects of the work in ensuring that questions related to the accuracy or integrity of any part of the work are appropriately investigated and resolved.

**HB:** Did data collection.
